# Primary Amebic Meningoencephalitis Related to Groundwater in Costa Rica: Diagnostic Confirmation of Three Cases and Environmental Investigation

**DOI:** 10.3390/pathogens9080629

**Published:** 2020-08-01

**Authors:** Lissette Retana Moreira, Leidy Zamora Rojas, Muriel Grijalba Murillo, Silvia Elena Molina Castro, Elizabeth Abrahams Sandí

**Affiliations:** 1 Departamento de Parasitología, Universidad de Costa Rica, San Pedro, Montes de Oca 11501, Costa Rica; lissette.retanamoreira@ucr.ac.cr; 2 Centro de Investigación en Enfermedades Tropicales (CIET), Universidad de Costa Rica, San Pedro, Montes de Oca 11501, Costa Rica; 3 Hospital Maximiliano Peralta Jiménez, Caja Costarricense de Seguro Social, Cartago 30102, Costa Rica; lmzamorar@ccss.sa.cr; 4 Laboratorio Clínico Hospital Enrique Baltodano, Caja Costarricense de Seguro Social, Liberia, Guanacaste 50101, Costa Rica; mgrijalb@ccss.sa.cr; 5 Instituto de Investigaciones en Salud (INISA), Universidad de Costa Rica, San Pedro, Montes de Oca 11501, Costa Rica; silvia.molinacastro@ucr.ac.cr

**Keywords:** Primary amoebic meningoencephalitis, *Naegleria fowleri*, hot springs, water well, Costa Rica

## Abstract

During the first trimester of 2020, the Ministry of Health of Costa Rica reported the first three cases of primary amebic meningoencephalitis (PAM). In two cases, laboratory personnel of the hospitals preliminarily identified amoeboid forms in cerebrospinal fluid (CSF) samples. For the molecular confirmation of species, CSF samples were sent to our laboratory. We carried out microscopic analyses and exflagellation assays. Besides, samples were cultured in 2% casein hydrolysate medium and in non-nutrient agar plates supplemented with *Escherichia coli.* Finally, PCR and sequencing were employed for the molecular diagnosis and species identification. In all cases, the presence of *Naegleria fowleri* was confirmed. An environmental investigation to identify the possible infection sources was also performed. Water samples from hot springs and groundwater from an artisan well were collected and after filtration and culture in non-nutrient agar plates supplemented with *E. coli*, thermotolerance and exflagellation assays were carried out. For the positive samples, PCR and sequencing were performed, confirming the presence of *N. fowleri* in several water samples. The report of these cases and the possible association with hot springs has had a significant impact on the population and health authorities of Costa Rica.

## 1. Introduction

Primary amebic meningoencephalitis (PAM) is an acute disease of rapid progression of the central nervous system caused by the thermophilic free-living amoeba *Naegleria fowleri*. The infection usually occurs when water with the amoebae enters the nose and gains access to the brain, migrating along the olfatory nerves through the cribriform plate [1]. The incubation period varies from 2 to 15 days and death typically occurs 3–7 days after the onset of symptoms [2]. Infections with *N. fowleri* are fatal in more than 95% of cases and until 2016, more than 300 cases of PAM were reported worldwide [3].

Most PAM cases are associated with exposure to warm, untreated freshwater while participating in recreational water activities such as swimming and diving [1]. However, other types of water exposure include the use of contaminated tap water in neti pots for sinus irrigation and performing ritual nasal rinsing with contaminated tap water [1].

*N. fowleri* has three stages in its life cycle: trophozoites, cysts, and flagellate forms. The cysts and flagellate forms appear when the environmental conditions (temperature and nutrients’ availability) are adverse for the amoeba. The presence of the flagellate stage is distinctive of the genus *Naegleria* and regarding the clinical diagnosis, this feature is relevant as it allows for a differential diagnosis with other free-living amoebae like *Acanthamoeba* and *Balamuthia*. *N. fowleri* trophozoites can tolerate temperatures of up to 45 °C, with an optimal temperature range between 30–42 °C [4]. This amoeba may proliferate in natural bodies of water like lakes and rivers during warmer months, as well as in hot springs and groundwater. Since the year 2000, a significant number of PAM cases involving groundwater as the possible source of infection have been reported; however, the isolation of the amoeba was possible only in a few of them [5].

**Figure 1:**
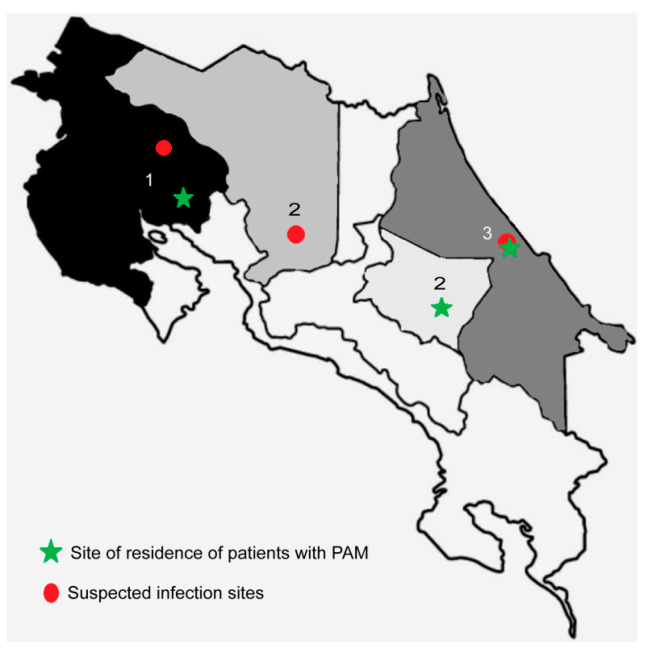
Provinces of residence of the three patients (1-2-3) diagnosed with primary amebic meningoencephalitis (PAM) in Costa Rica during January, February, and March, 2020 (marked with green stars). The suspected infection sites are also included (marked with red dots).

During 2020, three cases of PAM were diagnosed in Costa Rica. The cases presented in a 15-year old teenager (case 1), a 5-year old child (case 2), and in a 1-year old baby (case 3) from different provinces of the country (Figure 1). Of these cases, one patient successfully recovered from the infection after the treatment. Therefore, the objective of this study is to describe the methodology employed for the diagnostic confirmation of *N. fowleri* in these three cases. We also include an environmental investigation to identify the possible infection sources in two of the three cases.

## 2. Results

### 2.1. CSF Samples: Microscopic Analyses, Exflagellation Assays, Culture, and Molecular Confirmation of Species

**Figure 2:**
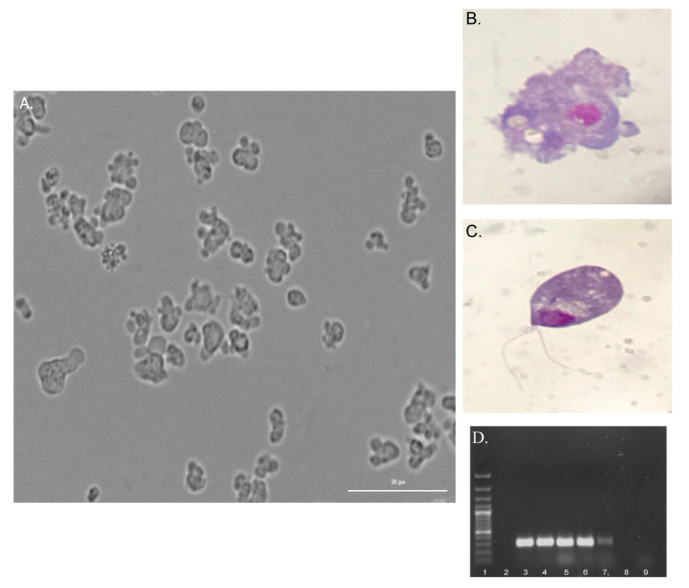
Microscopic analysis performed to CSF samples for the diagnosis of *N. fowleri*. In two of the three cases, trophozoites of active movement were identified in the CSF samples. The Figure shows trophozoites of *N. fowleri* in axenic culture using 2% casein hydrolysate culture medium (**A**) and Giemsa stains of the trophozoite (**B**) and the flagellate stage (**C**) of *N. fowleri.* (**D**) Molecular diagnosis of *N. fowleri* in CSF and water samples by PCR. Agarose gel electrophoresis after the amplification of the ITS rDNA of *N. fowleri* (NfITS primers) in some samples: (1) 1 Kb plus DNA ladder, (2) empty, (3) positive control of *N. fowleri,* (4–6) trophozoites after the axenic culture of the CSF samples of cases 1 and 3, (7) environmental sample from one water spring, (8) empty, (9) negative control.

CSF samples from three patients with suspected primary amoebic meningoencephalitis that presented in three consecutive months of 2020 were analyzed. In two of the three cases, the microscopic examination revealed the presence of numerous trophozoites of active movement due to wide pseudopodia (lobopod-type) of rapid formation (Figure 2 and Supplementary Video S1). The morphology observed and the positive exflagellation results suggested *Naegleria* (Figure 2). In these cases, cultures of the CSF samples were positive and amoebae are still maintained in 2% casein hydrolysate culture medium. For case 2, trophozoites were not observed during the microscopic analysis and cultures were negative.

PCR and sequencing were carried out for the species confirmation and the results confirmed the presence of *N. fowleri* in all cases. The real time PCR performed for the confirmation of case 2 was also positive. The nucleotide sequences were deposited in the GenBank database under the accession numbers MT090627, MT586316, and MT210902.

A video that shows the movement of the trophozoites is included in the Supplementary material.

### 2.2. Environmental Investigation: Analysis of Water Samples

As part of the epidemiological investigation of the cases, the Ministry of Health of Costa Rica requested the sampling and analysis of water from the suspected sources of infection related to cases 1 and 3 from our laboratory (Figure 1). The sampling included nine swimming pools and three water springs that served water to a resort visited by the patient of case 1 and six water samples from an artisan well and from the main water supplies for the house of the patient of case 3. For these cases, growth at 44.5 °C and exflagellation assays were positive in one swimming pool and two water springs related to case 1 and in one water sample from the piping outside the house of the patient of case 3. For case 2, the environmental study was not requested. The presence of *N. fowleri* in the aforementioned water samples was also confirmed by PCR (Figure 2) and sequencing. Besides, *N. lovaniensis* and *Vermamoeba vermiformis* were also identified in other water samples. The sequences were also deposited in the Genbank database under the accession numbers MT090626, MT210903, MT755633, MT755632, and MT586132.

## 3. Discussion

Epidemiologic studies of PAM indicate an estimate of 300–440 cases worldwide [3,6], with more than one third of the cases diagnosed in the United States [7]. However, this number may not exactly reflect the real number as some researchers propose an underestimation of the disease of up to 50% in this country, even when using well-established diagnostic methodologies and researchers with huge knowledge in different aspects related to PAM. The recent increase in the number of diagnosed cases outside the US suggests that PAM should be considered an emerging infectious disease [8]. According to the authors, most PAM cases have occurred in regions with warm temperatures and more than 90% are related to water exposure.

In this work, we performed the diagnostic confirmation of the first three cases of PAM that occurred in Costa Rica and in two of the three cases, we established an association with the possible infection sites. A history of aquatic activity or water exposure before the onset of symptoms and admission to hospital is reported in all cases. Furthermore, incubation periods of 2–7 days after the exposure to potentially contaminated water were presented, information that is consistent with previous reports in the literature [2]. For cases 1 and 3, *N. fowleri* was identified in the CSF samples and we successfully accomplished the axenic culture of the amoebae. In both cases, abnormalities in the cell counts, protein, and glucose levels in the CSF samples were consistent with PAM. Regarding case 2, the parameters of the CSF analysis were within the normal range, as informed by the laboratory personnel of the hospital, and the microscopic analysis did not reveal the presence of amoeboid forms. However, under clinical and epidemiologic suspicion, the pediatrician who was managing the case decided to ask for the differential diagnosis with *N. fowleri.* In this specific case, both conventional and real time PCRs confirmed the presence of the amoeba.

The three cases we report in this study are the first PAM cases described and fully diagnosed in Costa Rica, a country that maintains temperatures of 18–32 °C throughout the year and in most of its territory due to its geographical location. These temperatures are favourable for the optimal growth of the *N. fowleri*.

For the first two cases, health authorities suspected that the infection sources could be the hot springs that the patients visited 2–14 days before the onset of symptoms [9]. For the third case, suspicion fell on the groundwater from an artisan well that was employed for several activities by the family members (including bathing).

The environmental studies conducted for the first case demonstrated the presence of *N. fowleri* in the thermal springs and in one pool of the resort that the teenager visited. This is the second time that the country has reported this amoeba in hot springs. The first report corresponds to the finding of *N. fowleri* in hot springs in San Carlos, a report that was associated with a fatal case of PAM in an 11-year old boy who was a resident of Florida [10]. In this case, the clinical diagnosis was performed in the United States when the family returned home after vacationing in Costa Rica.

For the past several decades, thermal waters in Costa Rica have been considered important tourist attractions. The commonly called “geotourism” has been exploited in an accelerated way in regions near the Arenal volcano (in La Fortuna, San Carlos, Alajuela) and more recently, near the Rincón de la Vieja and Miravalles volcanoes and national parks in Guanacaste [11]. In the hotels and resorts of the latter region, children and young adults can practice aquatic activities such as diving and playing in water slides of different heights and lengths, which run into swimming pools of warm water (26–30 °C). These activities normally imply the entry of water through the nose, which constitutes a risk factor for infection with *N. fowleri*. In our study, for case 1, it is suggested that the infection may have occurred when the teenager submerged in the swimming pool or slid on the water slide that ran into the pool, removing the sediment from the bottom and increasing the water pressure towards his nose, causing the entry of the amoeba.

Regarding case 3, the analysis conducted by our laboratory demonstrated the presence of *N. fowleri* in samples of water derived from an artisan well. Additionally, *N. lovaniensis* and *Vermamoeba vermiformis* were also identified in two other water samples. The location where the sampling was performed corresponds to one of the lowest income communities in the area, with limited access to public services, including the lack of access to potable water. Around 89% of the families are supplied with untreated water from artisan wells 2–6 m deep [12]. Using an electric pump, the water is carried through piping to the houses, where it is kept inside plastic containers located in structures constructed at a 3 m height. This water is employed for cleaning, washing, and for daily baths. In 2016, microbiological and physicochemical analyses demonstrated the presence of fecal coliforms in 40% of the water samples from the wells of this region, in addition to a high concentration of manganese (mean: 0.87 mg/L) [12]. This value exceeded the 0.05 mg/L manganese limit stipulated in the regulation of the quality of potable water in Costa Rica [13] as well as the limit defined by the Environmental Protection Agency of the United States [14]. In addition to the toxic effects, high concentrations of this metal have been associated with a greater presence and proliferation of *N. fowleri* [15]. For example, concentrations of up to 20 mg/L manganese increase the growth rate of free-living amoeba such as *Acanthamoeba*, *N. fowleri*, and *Balamuthia* [16], while concentrations higher than 24 mg/L are toxic to *Acanthamoeba* [17]. However, according to Maciver et al., even when the presence of *N. fowleri* has been demonstrated in groundwaters, it is not yet clear how other microorganisms and physicochemical features could favor the presence and multiplication of this amoeba [8]. In this case, the finding of thermophilic amoebae could support the idea that the infection of the child occurred after the entry of this agent through his nose when his parents were bathing him, which could have occurred 2–5 days before the manifestation of the first symptoms. Although this case could not be strictly considered as transmission of *N. fowleri* through tap water, it should alert the health authorities and researchers to consider this type of water supply (artisan wells) as possible infection sources. It is important to highlight that collecting water from artisan wells by people is a common practice and the only available alternative to obtain water in some of the poorest communities in the world.

For cases 1 and 3, further studies to determine and compare the genotypes of the clinical and environmental isolates of *N. fowleri* could be performed, as described by other authors [1,18]. However, it is important to consider that current *N. fowleri* genotyping tools are not sufficient for molecular epidemiology purposes due to the lack of discriminatory power to definitively link the clinical and environmental isolates, as stated by Cope et al. [1].

The report of these cases of PAM in Costa Rica and the possible association with hot spring sources in two of them have had a significant impact on the population and health authorities of the country. The Ministry of Health is currently working on a modification of the regulation for the management of swimming pools that will include a specific section of guidelines for the use of natural hot springs, considering that *N. fowleri* is a waterborne pathogen that should be considered [19,20]. Some of the new conditions that the establishments must accomplish are: i) warning signs that indicate the risk of amoebic meningoencephalitis and ii) the prohibition of water slides or trampolines that discharge into natural hot springs [20]. Although, developing health awareness messages for the population is challenging because people are often exposed to *N. fowleri* but infections are rare [18]. The public has also been encouraged to take actions to reduce the risk of infection, such as the use of nose clips for swimming and diving, or to keep their head above the water while enjoying the hot springs.

## 4. Materials and Methods

### 4.1. History of the Cases

Cerebrospinal fluid samples of the following three suspected cases of PAM were transported to the laboratory of Medical Protozoology of the Department of Parasitology (University of Costa Rica, San Pedro, Montes de Oca) for the diagnostic confirmation and species identification.

#### 4.1.1. Case 1 

The first case corresponds to a 15-year old male that was admitted to a hospital in the province of Guanacaste (the northwestern region of Costa Rica) (Figure 1) in January 2020 [9]. He presented with general discomfort, strong headaches, nausea, and vomiting, so encephalitis of rapid progression was suspected. After 6 days of hospitalization, the patient died. According to the Ministry of Health, on initial suspicion of PAM, the patient’s family was asked about possible water exposures during the two weeks prior to the onset of symptoms. They stated that 7 days before the admission, the teenager visited a hot spring resort with family and friends [9]. No additional aquatic activity such as swimming, diving, or any recreational activity was reported during this time period.

#### 4.1.2. Case 2 

The second case corresponds to a 5-year old child who was taken to a hospital in the province of Cartago (in the central-eastern region of Costa Rica) (Figure 1) in February 2020 [20]. She presented lower limb pain, spasticity, hyperreflexia, and walking difficulty. Her parents stated that the previous day she presented with headaches, vomiting, and fever. They also indicated a visit to a hot spring resort in the province of Alajuela only two days before the onset of symptoms. Under clinical and epidemiologic suspicion and considering the recent case of PAM in the country, the pediatrician who was managing the case decided to start the treatment immediately, initially using amphotericin B administered by intravenous infusion. Twelve hours after starting the treatment, a CSF sample was obtained and analyzed. After 28 days of treatment, following the recommendations of the Centers for Disease Control and Prevention (CDC), the child fully recovered from the infection without apparent sequels. Due to the exceptional nature of this case, it will be thoroughly discussed in an article that is currently in progress.

#### 4.1.3. Case 3 

The third case corresponds to a 1-year-old baby that was admitted to a hospital in the province of Limón (in the eastern part of the country along the coast of the Caribbean Sea) (Figure 1) in March 2020 [21]. According to the statements of personnel of the Ministry of Health of Costa Rica, three days before being admitted to the hospital, the baby showed signs of a febrile illness that worsened during the following days, showing drowsiness and an altered state of consciousness. CSF samples were sent to our laboratory the same day of admission to the hospital; the health authorities confirmed the baby´s death later that same day. In this case, no visits to swimming pools, hot spring resorts, lakes, rivers, or ponds were reported; the suspicion fell in the use of untreated groundwater from an artisan well for bathing the baby.

### 4.2. CSF and Water Samples

CSF from three patients with a presumptive diagnosis of PAM were sent to the Department of Parasitology of the University of Costa Rica for culture and molecular confirmation of species. At each health center, the samples were submitted to microscopic analysis, cell counts, and protein and glucose determinations. For cases 1 and 2, the BioFire FilmArray Meningitis/Encephalitis Panel™ (Bio Fire Diagnostics, Salt Lake City, UT, USA; bioMérieux, Craponne, France) was applied so viruses, bacteria, and fungi were discarded as causal agents.

As part of the epidemiological investigation of the cases, the Ministry of Health of Costa Rica requested the sampling and analysis of water from the suspected sources of infection related to two cases. For case 1, water samples of 5 L were collected from the swimming pools of the resort that the patient visited 11 days before his death. Besides, three springs that serve water to this and other resorts of the area were also included in the analysis (2–2.5 L each sample). The samples were transported at room temperature and processed immediately. For case 3, and following our recommendations for water sampling and transport, personnel from the Ministry of Health collected six water samples of 1 L from an artisan well, as well as from the main water supplies for different houses, including the one the patient inhabited. These samples were collected only twelve hours after the patients´death and were immediately shipped at room temperature to the University of Costa Rica, where the analyses were performed within 5 h after the water collection.

For case 2, the environmental study was not requested.

### 4.3. Microscopic Examination

Each CSF sample (approximately 1 mL) was transferred to 1.5 mL Eppendorf tubes and centrifuged at 2500× *g* for 5 min. After this time, the supernatant was collected in a new tube and the sediment was placed over a glass slide for the microscopic observation of the sample. Giemsa stains were also performed [22].

### 4.4. Sample Culture

#### 4.4.1. Axenic Culture

CSF samples with trophozoites were cultured in 25 cm^2^ Nunc™ EasYFlask cell culture flasks (Thermo Fisher Scientific, Waltham, MA, USA). The culture medium consisted of 2% casein hydrolysate (Merck-KGaA, Darmstadt, Germany) in distilled water, supplemented with 10% inactivated fetal bovine serum (Gibco, GranIsland, NY, USA). The flasks were incubated at 37 °C and amoebae were observed daily.

#### 4.4.2. Xenic Culture

The sample of CSF without the evident presence of trophozoites (case 2) was cultured over non-nutrient agar plates supplemented with *Escherichia coli*. The incubations were performed at 37 °C, with daily observation over 14 days.

#### 4.4.3. Culture of Water Samples

Water samples were filtered through 0.45 µm pore diameter nitrocellulose membranes (Merck-KGaA) and seeded onto non-nutrient agar plates supplemented with *E. coli*, as previously described [10]. These plates were incubated at 37 °C for at least 7 days. Amoebae morphologically similar to *Naegleria* were subcultured and incubated at 44.5 °C. Subcultures with positive growths at this temperature were submitted to the exflagellation assay.

### 4.5. Exflagellation Assays

CSF samples with trophozoites and water samples with amebae of similar morphology to *Naegleria* and positive growth at 44.5 °C were submitted to an exflagellation assay. Briefly, CSF samples were centrifuged as previously described and one drop of the sediment was placed in a 24-well plate with 1 mL sterile distilled water. The incubation was performed for 3 h at 37 °C.

For water samples, subcultures of amoebae with positive growths at 44.5 °C were flushed from the agar with 1 mL cold sterile PBS, transferred into 1.5 mL Eppendorf tubes, and centrifuged at 3000× *g* for 10 min. The supernatant was discarded and the sediment was placed in a 24-well plate with 1 mL sterile water. The incubation was performed as previously described.

### 4.6. DNA Extractions

DNA extraction of the CSF samples was performed directly from the sediment obtained after the centrifugation process. Besides, DNA extractions from trophozoites of two different positive cultures were also performed. Regarding water samples, the DNA extraction was performed from the sediment obtained after the centrifugation of the flushes of the agar plates with cold sterile PBS. DNA was purified using the QIAmp DNA Mini Kit (Qiagen, Hilden, Germany), following the manufacturers´ instructions.

### 4.7. PCR Amplification and Sequencing

The complete ITS region (ITS1, 5.8S, and ITS2) was amplified as previously described [23]. The nucleotide sequences of the Vahlkampfiids´ specific primers employed were as follows: Vahl-F: 5′-GTCTTCGTAGGTGAACCTGC-3′, VahlR: 5′-CCGCTTACTGATATGCTTAA-3′. Specific primers were also employed for the amplification of the ITS of *Naegleria* and *N. fowleri*, as previously described [23]. The nucleotide sequences of the primers employed are: the forward primer ITSFW 5´-AACCTGCGTAGGGATCATTT-3´ and the reverse primer ITSRV 5´- TTTCCTCCCCTTATTAATAT-3´ for *Naegleria* and the forward primer NfITSFW 5´-TGAAAACCTTTTTTCCATTTACA-3´ and the reverse primer NfITSRV 5´-AATAAAAGATTGACCATTTGAAA-3´ for *N. fowleri*. All amplification reactions were performed in a 50 µL mixture containing 50–100 ng/µL of genomic DNA and the PCR mix with the Taq buffer, 0.25 mM of each dNTP, 1 mM of each primer, and 1.25 U of the DreamTaq DNA polymerase (Thermo Fisher Scientific). Amplifications were run in a Biometra TOne thermal cycler (Labgene Scientific, Châtel-Saint-Denis, Switzerland), using the conditions previously described by Moussa et al. [24]. PCR products were purified using the QIAquick PCR purification kit (Qiagen) and sequenced in both directions at the “Centro de Investigación de Biología Celular y Molecular” (CIBCM) of the University of Costa Rica.

Negative DNA controls (template DNA replaced with distilled water), positive controls (*N. fowleri* Lee ATCC 30894 DNA), and DNA from the samples were analyzed using the three sets of primers except for one CSF sample (case 2), due to the low amount of DNA. In this case, besides conventional PCRs using the Vahlkampfiids´ specific primers and the ITS specific primers for *Naegleria*, an “in-house” RT-PCR was carried out with the species-specific NfITS primers. The reaction was performed using the Maxima SYBR Green/ROX qPCR Master Mix (Thermo Fisher Scientific) in a Rotor-Gene Q thermal cycler (Qiagen).

## Supplementary Materials

The following are available online at https://www.mdpi.com/2076-0817/9/8/629/s1, Video S1: Isolation of *Naegleria fowleri* from a CSF sample and culture in 2% casein hydrolysate medium. The videos were taken using the LyonHeart FX automated microscope (BioTek).
